# Evaluation of the Oxidative Effect of Long-Term Repetitive Hyperbaric Oxygen Exposures on Different Brain Regions of Rats

**DOI:** 10.1100/2012/849183

**Published:** 2012-02-02

**Authors:** Kemal Simsek, Mehmet Ozler, Ali Osman Yildirim, Serdar Sadir, Seref Demirbas, Muzaffer Oztosun, Ahmet Korkmaz, Hakan Ay, Sukru Oter, Senol Yildiz

**Affiliations:** ^1^Department of Undersea and Hyperbaric Medicine, Gulhane Military Medical Academy, 06010 Etlik, Ankara, Turkey; ^2^Department of Physiology, Gulhane Military Medical Academy, 06010 Etlik, Ankara, Turkey; ^3^Department of Emergency Medicine, Gulhane Military Medical Academy, 06010 Etlik, Ankara, Turkey; ^4^Department of Internal Medicine, Gulhane Military Medical Academy, 06010 Etlik, Ankara, Turkey; ^5^Health Service Command, Turkish Armed Forces, 06790 Etimesgut, Ankara, Turkey

## Abstract

Hyperbaric oxygen (HBO_2_) exposure affects both oxidative and antioxidant systems. This effect is positively correlated with the exposure time and duration of the treatment. The present study aims enlightening the relation of HBO_2_ with oxidative/antioxidant systems when administered in a prolonged and repetitive manner in brain tissues of rats. Sixty rats were divided into 6 study (*n* = 8 for each) and 1 control (*n* = 12) group. Rats in the study groups were daily exposed 90-min HBO_2_ sessions at 2.8 ATA for 5, 10, 15, 20, 30 and 40 days. One day after the last session, animals were sacrificed; their whole brain tissue was harvested and dissected into three different regions as the outer grey matter (cortex), the inner white matter and cerebellum. Levels of lipid peroxidation and protein oxidation and activities of superoxide dismutase and glutathione peroxidase were measured in these tissues. Malondialdehyde, carbonylated protein and glutathione peroxidase levels were found to be insignificantly increased at different time-points in the cerebral cortex, inner white matter and cerebellum, respectively. These comparable results provide evidence for the safety of HBO treatments and/or successful adaptive mechanisms at least in the brain tissue of rats, even when administered for longer periods.

## 1. Introduction

Hyperbaric oxygen (HBO_2_) therapy (HBOT) is the medical use of oxygen at a level higher than atmospheric pressure [1]. Its principle depends, at least in part, on the vital nature of oxygen needed to provide energy and support cellular respiration. It is obvious that decreased delivery of oxygen can affect cell survival. Several diseases or injuries can decrease the body's ability to transport oxygen to the tissues, increase the tissue demands for oxygen, and may elongate the distance that the oxygen must travel from the capillary to reach the cell [2]. Depending on this fact, HBOT has been successfully used in treating various pathological conditions underlying an inflammatory background [3], such as colitis [4], cystitis [5], pancreatitis [6], or sepsis [7, 8].

From another point of view, due to the large amounts of pure oxygen breathed, HBO_2_ treatments were also hold responsible for the potential of oxygen toxicity [9, 10]. In the medical literature, noticeable evidence has been accumulated proving that even a single HBO_2_ exposure can trigger oxidative stress [11–13]. Free radical generation, subsequently leading to oxidative stress, has long been known as at least one of the reasons of central nervous system (CNS) oxygen toxicity [14].

Apart from the former studies performed at supranormal pressure/duration ranges of HBO_2_ exposure, that is, higher than 3 atmospheres and longer than 2 hours, in order to test some protective agents against CNS oxygen toxicity [15–17], more recent works evidenced that, even within its approved therapeutic limits, HBO_2_ treatment enhances oxidative stress markers in brain tissue [18–20]. Since HBO_2_ applications have been mostly performed over a longer period with repetitive exposures [1], it is of particular importance to test its interactions with oxidative/antioxidant systems in experimental sets simulating its clinical use, for example, lower than 3 atm and 2 h.

In a recent study, we demonstrated a rise in lipid and protein oxidation products in the lung tissues of rats accompanied by increased antioxidant enzyme activities when HBOT was continued for more than 20 sessions [21]. Apart from the lung as being the entering site of hyperoxic injuries, the CNS is mainly accepted as another important target for oxygen exposure in toxic amounts [22]. Depending on this fact, the present study was conducted in order to enlighten the effects of consecutive HBO_2_ exposures from 5 up to 40 daily sessions on oxidative stress and antioxidant defense markers of rat's different brain regions.

## 2. Materials and Methods

### 2.1. Study Design

Our institutional Experimentation and Ethics Committee approved the experimental procedures of the study. A total of 60 adult male Sprague-Dawley rats bred in Gulhane Military Medical Academy Research and Progress Center were used. The rats were 12 weeks old and weighed 200–250 g at the beginning of the experiment. Housing was at 22–24°C with light from 08.00 AM to 08.00 PM and free access to water. All animals were fed standard commercial rat chow during the experiment.

Rats were divided into 6 study groups (*n* = 8 for each) which were exposed to HBO_2_ for 1, 2, 3, 4, 6, and 8 weeks. Weekly HBO_2_ administrations were set as 5 daily consecutive exposures followed by 2-day intervals. All animals in the study groups were sacrificed one day after their final HBO_2_ treatment in order to avoid possible interference of the acute postexposure phase. Separate control groups consisted of 6–8 animals for each time points were forbidden by our institutional Ethics Committee (issue 08/75 K). Thus, 2 control animals were sacrificed at the same time points with each of the 6 study groups (*n* = 12 in total for control group) in order to evaluate possible effects of aging. Details of the experimental schedule are to be seen in Table 1.

### 2.2. HBO_2_ Exposures

An animal hyperbaric chamber (made in Etimesgut Military Equipment Factory, Ankara, Turkey) was used for the HBO_2_ administrations. The HBO_2_ sessions were set as 2.8 atm pressure for 90 min in all study groups. Compression and decompression of the chamber was completed gradually in 5–10 min; continuous 100% O_2_ ventilation at a rate of 3-4 L/min was maintained throughout the 90 min exposure periods in the chamber. All administrations were started at the same hour in the morning (10.00 AM) to equalize possible effects of the circadian rhythm [23].

### 2.3. Tissue Preparation

For tissue sampling, animals were anesthetized (i.p. ketamine + xylazine, 85 + 12.5 mg/kg) 24 h after the last HBO session. Their skulls were opened, and their whole brain tissues were harvested. Then the brain tissues were immediately dissected into its three different regions as the outer grey matter (cerebral cortex), the inner white matter, and the cerebellum, put into tubes, and frozen with liquid nitrogen. The frozen tissues were homogenized in phosphate buffer (pH 7.4) by means of a homogenizer (Retsch Mixer Mill MM 400: Düsseldorf, Germany) and centrifuged (Hermle Z323K: Gosheim, Germany) at 2,500 rpm for 10 min. The supernatants were divided into two to three parts, put in separate tubes, and stored at −80°C until assay.

### 2.4. Biochemical Analysis

The supernatants of the tissue homogenates were used for the entire assays. Lipid peroxidation levels were measured using the thiobarbituric acid reaction by the method of Ohkawa et al. [24]. This method was used to obtain a spectrophotometric (Helios epsilon, USA) measurement of the color produced during the reaction to thiobarbituric acid with malondialdehyde (MDA) at 535 nm. Tissue protein carbonyl content (PCC) was determined with the method described by Levine et al. [25]. Final calculated MDA and PCC levels were expressed as millimoles per gram protein. The activity of the antioxidant enzyme superoxide dismutase (SOD) was assayed using the nitroblue tetrazolium (NBT) method of Sun et al. [26]. Briefly, NBT was reduced to blue formazan by the superoxide anion radical, which has strong absorbance at 560 nm. One unit (U) of SOD is defined as the amount of protein that inhibits the rate of NBT reduction by 50%. Glutathione peroxidase (GSH-Px) activity was measured using the method described by Paglia and Valentine [27]. The estimated SOD and GSH-Px activities were expressed as units per gram protein. Finally, in order to standardize the measured data, the protein content of the hemolysates was measured according to the method of Lowry et al. [28] with bovine serum albumin as the standard.

### 2.5. Statistical Analyses

Normality analyses were performed by using the Kolmogorov-Smirnov and Shapiro-Wilk tests, and the entire data of the study was found to be normally distributed. Thus, parametric statistics were used for the evaluation of the results; that is, if the *one way analysis of variance (ANOVA)* indicated intergroup significance, the post hoc *Bonferroni* test was performed for group-to-group comparisons. *P* values less than 0.05 were considered significant. All analyses were performed using the SPSS software (Version 15.0; SPSS, Chicago, IL, USA).

## 3. Results

According to general observations, symptoms for barotraumas, hyperoxic convulsions, and weight gain or loss, no unexpected or adverse effects were observed throughout the experimental period. All animals survived the study period until being sacrificed for tissue sampling and analyzing. None of the measured parameters revealed any significant change when compared with the control values (*P* > 0.05 for all parameters at all measure points versus their related control group). The entire data of the study is presented in box-plot graphics showing the median, minimum, maximum values, and the quartiles for each group.

### 3.1. Brain Cortex Tissue (Grey Matter)

**Figure 1:**
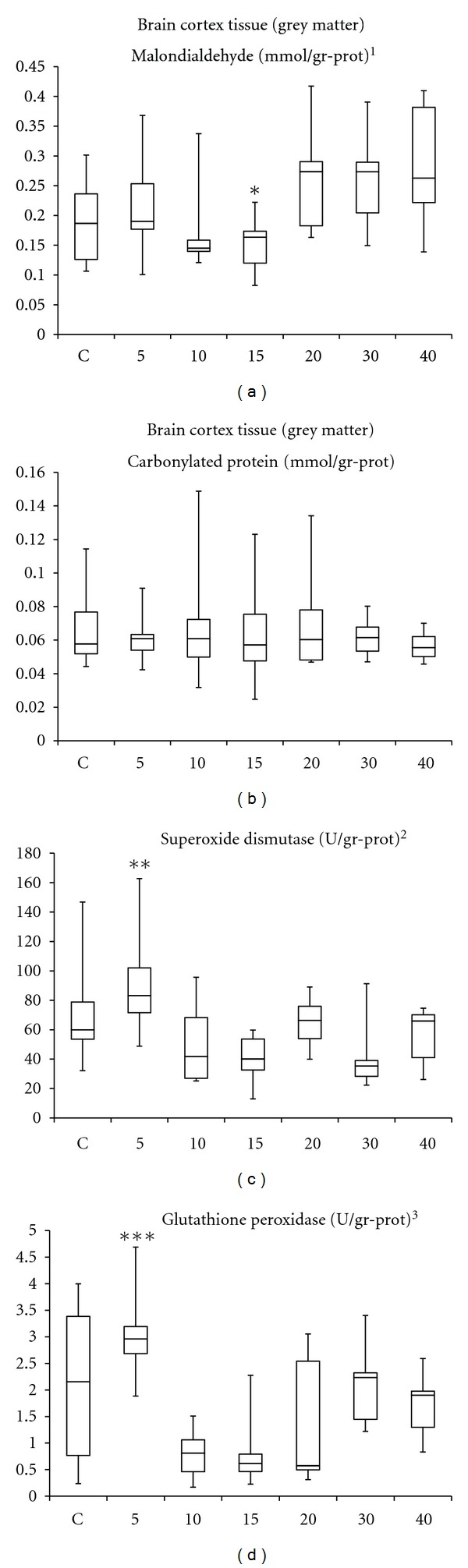
Oxidation products and antioxidant enzymes in brain cortex tissue. C: control group; numbers on the *x*-axis stand for the HBO_2_ session number of the groups. *One-way ANOVA*: **^1^** *P* = 0.016; **^2^** *P* = 0.005; **^3^** *P* < 0.001. *Bonferroni*: *P = 0.047 versus 40-session group; **P = 0.013 versus 15-, and *P* = 0.012 versus 30-session groups; ***P < 0.001 versus 10-, *P* = 0.001 versus 15-, and *P* = 0.043 versus 20-session HBO_2_ groups. Note that no significant changes for any measured parameters were recorded in comparison with the control values.

In cortex tissue, MDA values tended to increase with longer HBO_2_ exposure periods of 20, 30, and 40 sessions, but this slight increase was estimated to be statistically insignificant. No evidence for protein oxidation (PCC) and no evident changes in the antioxidant enzymes SOD and GSH-Px activities were recorded in brain cortex specimens in comparison with control levels (*P* > 0.05). Group-to-group comparisons revealed significantly increased MDA values in 40-session HBO_2_ group versus the 15-session group, significantly decreased SOD activities in the 15- and 30-session groups versus the 5-session group, and significantly decreased GSH-Px activities in the 10-, 15-, and 20-session groups versus the 5-session HBO_2_ exposure group (*P* < 0.05; Figure 1).

### 3.2. Brain Inner Material (White Matter)

**Figure 2:**
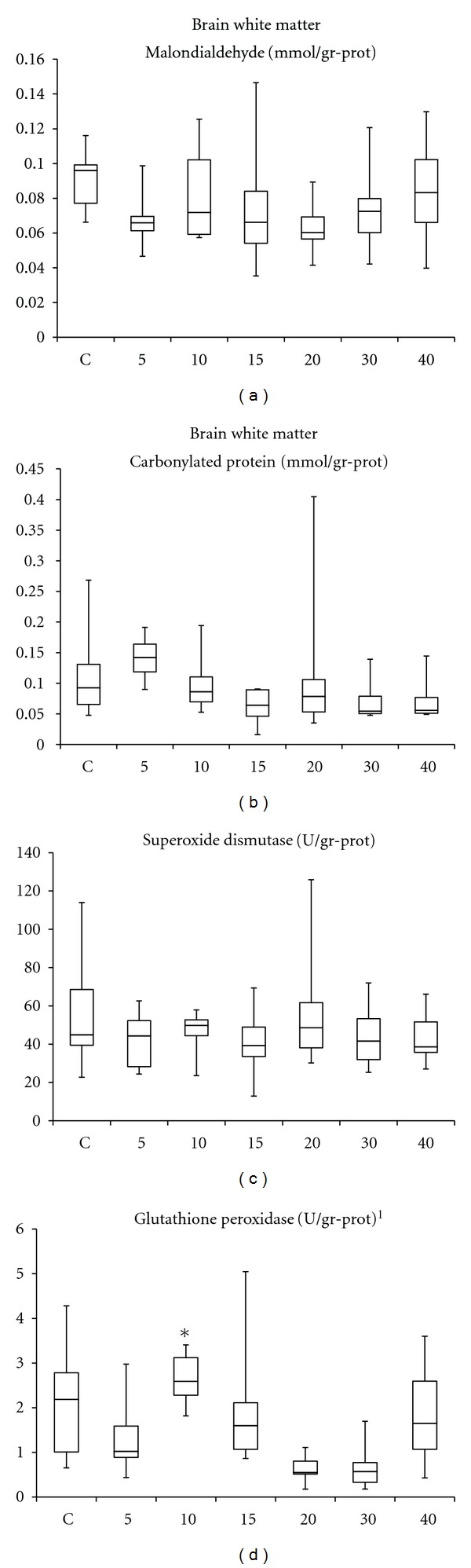
Oxidation products and antioxidant enzymes measured in the white matter of the brain. C: control group; numbers on the *x*-axis stand for the HBO_2_ session number of the groups. *One-way ANOVA*: **^1^** *P* = 0.001. *Bonferroni*: *P = 0.005 and *P* = 0.006 versus 20-, and 30-session HBO_2_ groups, respectively. No significant variations compared with the control group were recorded.

The white matter of the brain presented no decisive change for MDA values or SOD and GSH-Px activities compared to controls (*P* > 0.05). Only a light insignificant increase of PCC with just 5 HBO exposures was to be seen. Apart from the control group, the 20- and 30-times HBO_2_ exposed groups presented significantly lower GSH-Px activities than the 10-session group (*P* < 0.05; Figure 2).

### 3.3. Cerebellum

**Figure 3:**
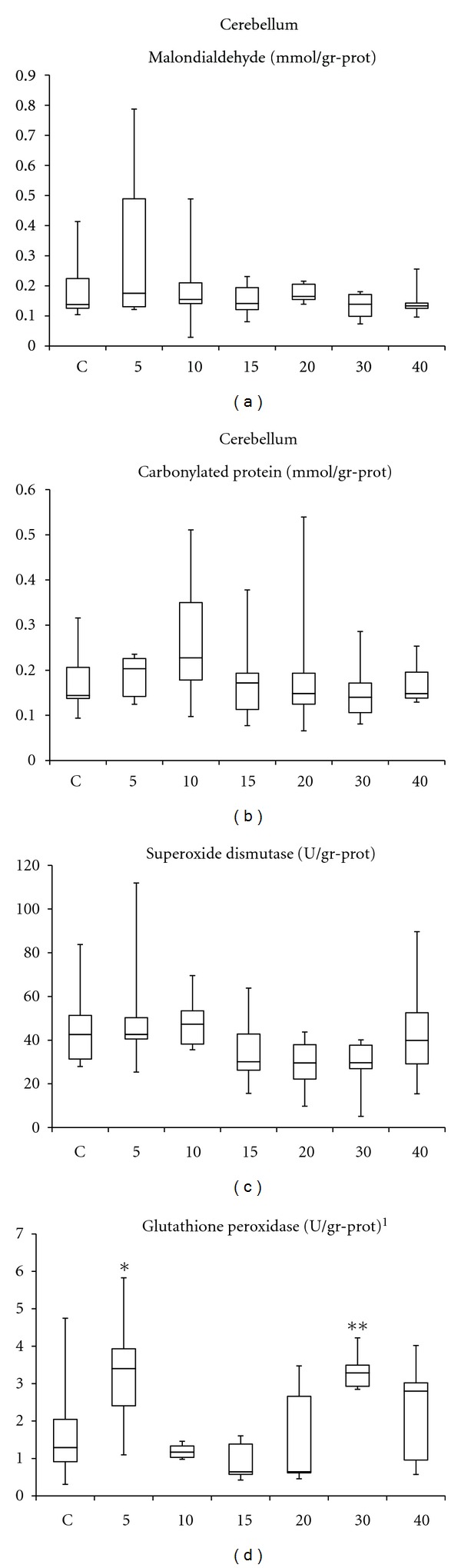
Cerebellar values of oxidation products and antioxidant enzymes activities. C: control group; numbers on the *x*-axis stand for the HBO_2_ session number of the groups. *One way ANOVA*: **^1^** *P* < 0.001. *Bonferroni*: *P = 0.009 and *P* = 0.004, **P = 0.01 and *P* = 0.004 versus 10- and 15-exposure groups, respectively. Comparisons with the control groups resulted in comparable values.

In the 5- and 30-session HBO_2_ exposure groups, increased levels of GSH-Px activities were recorded; however, due to the wide distribution of the in-group data, these levels were also not statistically significant. Cerebellar SOD activities, MDA, and PCC values remained nearly unchanged at each measure point (*P* > 0.05). Detailed comparisons indicated that the GSH-Px activities of the 10- and 15-HBO_2_ exposure groups were significantly less than the 5- and 30-session groups (*P* < 0.05; Figure 3).

## 4. Discussion

In the present study, the oxidative potential as well as the CNS oxygen toxicity risk of daily repetitive HBO_2_ exposures over a prolonged period was tested in rat's brain tissue. The main two outcomes of this study were that (i) with regard to lipid and protein oxidation product's values, HBO_2_ administrations from a minimum of 5 and up to 40 sessions caused no oxidative stress in different brain tissue regions of the rats and (ii) antioxidant enzymes SOD and GSH-Px activities remained also nearly unchanged throughout the same experimental set.

Earlier works of our institutional study group designated a clear oxidative effect of acute one-session HBO_2_ exposures in rat's brain cortex tissue [18–20]. These studies indicated a pressure- [18] and exposure-time- [19] related oxidative action by measuring lipid peroxidation products in brain cortex homogenates. The maximal HBO_2_ exposure time and pressure in the above-mentioned works were limited with the maximal approved clinical used limits as 2 h and 3 atm [1], respectively; however, the evidenced oxidative effect began just with the halves of these limits, that is, 1 h and 1.5 atm. The unchanged levels of the oxidative stress markers MDA and PCC in the present study appear to be contradictory when compared with the former experiments. On the other hand, in previous studies it was also seen that the oxidative effect after a single HBO_2_ exposure, even at its maximal approved safe limits of 3 atm for 2 h, remains no more than 90 min after removing the animals from the hyperbaric chamber [20]. As described previously, in the current work the animals were sacrificed 24 h after the last HBO_2_ administrations; this may be one explanation for the present outcome.

When experimental animals were exposed to HBO_2_ at supranormal amounts, that is, above 3 atm and 2 h, the main outcome was a clear increase in biooxidative products combined by an exhaustion of endogen antioxidants, namely, settled oxidative stress [29, 30]. Nevertheless, if the HBO_2_ administration process was set within therapeutically used and approved limits, the antioxidant levels mostly accompany the rise of oxidation products [18–20, 31, 32]. Different from these reports and similar to their outcome for oxidation products, the current work represents no significant changes for SOD and GSH-Px activities in three different regions of the rat's brain. Again, the 24 h waiting period after the final HBO_2_ session may be the reason for this finding.

A more recent work of our team, conducted with the same repetitive HBO_2_ exposure procedure of the current one, resulted in increased oxidative stress markers and antioxidant enzymes after 20 and more sessions in rat's lung tissue [21]. The different nature of the response of lung and brain tissues, two main targets of hyperoxic hyperoxia [18, 29], is quite interesting and needs to be clarified by further research. The present limited outcome can just be interpreted as a sign for a more efficient defense system of the CNS than the lung, or a gradually lessened toxicity of oxygen depending on the distance from the primarily attacked lung cells to the brain.

In the medical literature, there are former reports about an adaptive mechanism which protects against further oxidative damage when HBO_2_ was administered for more than a single exposure [33, 34]. Other reports manifested HBOT as a beneficial preconditioning application in order to prevent several organs or tissues from following oxidative injuries [35, 36]. These adaptive and preconditioning actions triggered by HBO_2_ treatments may also be responsible for the underlying mechanisms of the present findings. The intracellular antioxidant enzyme heme oxygenase-1 (HO-1) has mainly been hold responsible for the HBOT-caused adaptive changes [36, 37]; however, with the limited parameters of the present study, we cannot prove whether this molecule is the key factor for unchanged oxidative stress markers even after 40 HBO_2_ exposures or not.

In a previous study conducted in our institution, increased PCC values and SOD activities were recorded in rat's lung and brain tissues after a 10-session HBO_2_ exposure, each at 2.5 atm for 1 h daily [32]. The main difference of the present study from this one is the time of tissue harvesting; in the previously mentioned work the animals were sacrificed immediately after the last HBO_2_ treatment but, as emphasized previously, in the present one animals were hold for one day until their brain tissues were collected. This result obviously designates to a number of biological arrangements resulting in a more effective scavenging action against HBO_2_-induced oxidative attack during the 24 h resting period afterwards.

The first sign of CNS oxygen toxicity has been described to be the hyperoxic seizures/convulsions [38]. A considerable number of experimental works have long been concentrated on the underlying mechanisms of this reaction [15–17, 39–41]. Interestingly, some of these studies reported that repeated exposures to HBO_2_ increased the sensitivity to seizures with regard to free radical and/or nitric-oxide-dependent mechanisms [42, 43]. However, our present study revealed no sign for an increased production of HBO_2_-induced free radical production or subsequent oxidative effects on biomolecules with repeated exposures. As a possible reason, it must be taken into consideration that all of the previously mentioned studies were conducted at extreme high pressure varying from 4 to 7 atm and were straightly directed to induce CNS toxicity in order to examine the reasons or preventive methods; on the other side, the present work is set at clinically approved pressure/duration ranges, and therefore it is not unpredicted to result without any toxicity sign.

Since HBO_2_ is an important therapeutic approach with life-saving properties in various conditions and its efficacy generally depends on repeated exposures for several days [1], it is of particular importance to define its molecular interactions when administered in repetitive manner. Different from the previous study which revealed some oxidative actions in the lung tissue after 20 and more HBO_2_ sessions [21], the present work resulted in nonsignificant changes of oxidative and antioxidant system markers in brain tissue with up to 40 consecutive HBO_2_ exposures. This result may be interpreted as a sign for (i) a robust defensive mechanism against the hyperoxic attack, (ii) an adaptive response as reported in earlier studies [33, 34], and/or (iii) an effective repair mechanism scavenging the entire injury within the 24 h postexposure period in CNS of the rats. Further studies, concentrated on transcription factors and their target genes known to be triggered and activated with HBOT may help to elucidate the exact pathways and molecular interactions which occur during or after repeated HBO_2_ administrations.

## Figures and Tables

**Table 1:** Experimental schedule of the entire study.

	*n*	Mon.	Tue.	Wed.	Thu.	Fri.	Sat. (sacrificing)	Sun.
Week 1	48 HBO_2_ + 12 C	HBO_2_	HBO_2_	HBO_2_	HBO_2_	HBO_2_	8 HBO_2_ + 2 C	N/A
Week 2	40 HBO_2_ + 10 C	HBO_2_	HBO_2_	HBO_2_	HBO_2_	HBO_2_	8 HBO_2_ + 2 C	
Week 3	32 HBO_2_ + 8 C	HBO_2_	HBO_2_	HBO_2_	HBO_2_	HBO_2_	8 HBO_2_ + 2 C	
Week 4	24 HBO_2_ + 6 C	HBO_2_	HBO_2_	HBO_2_	HBO_2_	HBO_2_	8 HBO_2_ + 2 C	
Week 5	16 HBO_2_ + 4 C	HBO_2_	HBO_2_	HBO_2_	HBO_2_	HBO_2_	N/A	
Week 6	16 HBO_2_ + 4 C	HBO_2_	HBO_2_	HBO_2_	HBO_2_	HBO_2_	8 HBO_2_ + 2 C	
Week 7	8 HBO_2_ + 2 C	HBO_2_	HBO_2_	HBO_2_	HBO_2_	HBO_2_	N/A	
Week 8	8 HBO_2_ + 2 C	HBO_2_	HBO_2_	HBO_2_	HBO_2_	HBO_2_	8 HBO_2_ + 2 C	

All of the HBO_2_ administrations and animal sacrificing were performed at 10.00 AM (C: control *n*: animal count).
